# Modulation of Inflammation and Immune Responses by Heme Oxygenase-1: Implications for Infection with Intracellular Pathogens

**DOI:** 10.3390/antiox9121205

**Published:** 2020-11-30

**Authors:** Diego L. Costa, Eduardo P. Amaral, Bruno B. Andrade, Alan Sher

**Affiliations:** 1 Departamento de Bioquímica e Imunologia, Faculdade de Medicina de Ribeirão Preto, Universidade de São Paulo, Ribeirão Preto 14049-900, São Paulo, Brazil; 2 Immunobiology Section, Laboratory of Parasitic Diseases, National Institute of Allergy and Infectious Diseases, National Institutes of Health, Bethesda, MD 20892, USA; eduardo.amaral@nih.gov (E.P.A.); asher@niaid.nih.gov (A.S.); 3 Wellcome Centre for Infectious Disease Research in Africa, Institute of Infectious Disease and Molecular Medicine, University of Cape Town, Cape Town 7925, South Africa; bruno.andrade@fiocruz.br; 4 Instituto Gonçalo Moniz, Fundação Oswaldo Cruz, Salvador 40296-710, Bahia, Brazil; 5 Multinational Organization Network Sponsoring Translational and Epidemiological Research (MONSTER) Initiative, Salvador 40210-320, Bahia, Brazil; 6 Curso de Medicina, Faculdade de Tecnologia e Ciências (UniFTC), Salvador 41741-590, Bahia, Brazil; 7 Curso de Medicina, Universidade Salvador (UNIFACS), Laureate International Universities, Salvador 41770-235, Bahia, Brazil; 8 Escola Bahiana de Medicina e Saúde Pública (EBMSP), Salvador 40290-000, Bahia, Brazil; 9 Division of Infectious Diseases, Department of Medicine, Vanderbilt University School of Medicine, Nashville, TN 37232, USA

**Keywords:** heme oxygenase-1, inflammation, immune response, infectious disease, intracellular pathogens, host directed therapy

## Abstract

Heme oxygenase-1 (HO-1) catalyzes the degradation of heme molecules releasing equimolar amounts of biliverdin, iron and carbon monoxide. Its expression is induced in response to stress signals such as reactive oxygen species and inflammatory mediators with antioxidant, anti-inflammatory and immunosuppressive consequences for the host. Interestingly, several intracellular pathogens responsible for major human diseases have been shown to be powerful inducers of HO-1 expression in both host cells and in vivo. Studies have shown that this HO-1 response can be either host detrimental by impairing pathogen control or host beneficial by limiting infection induced inflammation and tissue pathology. These properties make HO-1 an attractive target for host-directed therapy (HDT) of the diseases in question, many of which have been difficult to control using conventional antibiotic approaches. Here we review the mechanisms by which HO-1 expression is induced and how the enzyme regulates inflammatory and immune responses during infection with a number of different intracellular bacterial and protozoan pathogens highlighting mechanistic commonalities and differences with the goal of identifying targets for disease intervention.

## 1. Introduction

Heme oxygenases (HOs) are highly conserved enzymes that are present in a large number of species as evolutionary distant as algae and humans [1]. The mechanisms associated with the catabolism of heme by the heme oxygenase system were first demonstrated in 1968 by Tenhunen et al. utilizing microsomes from rat livers [2]. HO itself was first purified in 1977 by Maines et al. [3] and almost 10 years later, in 1986, the same author identified two isoforms of the enzyme in rat liver microsomes: heme oxygenase-1 (HO-1), whose expression in liver was upregulated in response to in vivo treatment of rats with chemicals, and heme oxygenase-2 (HO-2), whose constitutive expression remained unaltered by in vivo challenge with the same compounds [4]. In parallel, different groups identified a 32 kDa stress-responsive protein, whose expression was highly upregulated in different cell lines in response to challenges such as hyperthermia, heavy metals and oxidative stress. This protein, referred to as Heat-Shock Protein 32 (HSP-32) at that time [5,6,7,8,9,10], was later shown to be identical to HO-1 [11,12]. A third isoform, heme oxygenase-3 (HO-3), was characterized in 1997 in a number of different rat tissues, but displayed poor enzymatic activity [13] and its physiological role remains largely uncharacterized to this day. A later study in 2004 failed to isolate rat HO-3 protein and questioned the existence of its gene speculating that the originally identified HO-3 may in fact represent the product of pseudogenes derived from HO-2 transcripts [14].

The main function of HO-1 and HO-2 is to catalyze the degradation of heme molecules, providing the rate-limiting step for this reaction, which consumes three molecules of O_2_ and seven electrons resulting in the release of equimolar amounts of carbon monoxide (CO), ferrous iron (Fe^2+^) and biliverdin [15]. Both HO-1 and HO-2 have a conserved heme catalytic domain, while HO-2 has two additional heme-binding regions, which are associated with regulation of its activity, but are devoid of enzymatic function [16]. It has recently been determined that these regions play a role in the transfer of heme molecules to the catalytic site [17]. After heme binds to HO, the first step of the degradation reaction uses an O_2_ molecule and electrons donated by NADPH–cytochrome-P450-reductase to oxidize heme into α-meso-hydroxyheme, releasing a water molecule. α-meso-hydroxyheme further reacts with another oxygen molecule resulting in the release of verdoheme and CO. A subsequent reaction step consumes a third O_2_ molecule and again electrons donated by NADPH–cytochrome-P450-reductase, converting verdoheme into biliverdin and ferrous iron [18].

In homeostatic conditions, HO-1 is detected mainly in the liver and spleen, while HO-2 is stably expressed in most organs with higher levels being found in testes, spleen, liver, kidney, cardiovascular and central nervous systems [19,20]. Unlike HO-2, whose expression remains unchanged after challenge with different stressors [16], HO-1 expression is induced in cells from most tissues in response to oxidative stress and inflammation and is particularly highly upregulated in macrophages affected by infection with intracellular microorganisms [21,22].

Heme and the products of its degradation can influence the outcome of infection with intracellular pathogens in different ways. Heme can be used as a source of nutrient iron for replication by intracellular pathogens, directly enhancing their survival and growth inside their host cell niches including phagocytes [23]. On the other hand, the CO and bilverdin products of heme degradation can exert both pro and anti-inflammatory roles, resulting in the suppression of immune responses and control of pathogen replication, or in the regulation of pathogenesis and inflammation-driven tissue damage [24,25]. In the center of this process lies the inducible HO-1 isoform, which by mediating heme degradation can markedly influence the outcome of infection with intracellular pathogens through the diverse effects of it enzymatic products [26]. Due to its close association with the inflammatory process, numerous studies have evaluated the use of HO-1 as a biomarker for severity or prognosis of such infectious diseases. This work has been accompanied by mechanistic studies examining the role of HO-1 in both host resistance and pathogenesis employing genetically deficient animals or pharmacological inhibitors or inducers of the enzyme’s activity or expression as investigative tools. Here we will review current knowledge of the mechanisms that control the enzyme’s expression in homeostasis and inflammation and how in turn, HO-1 can regulate inflammatory and immune responses. Finally, we will discuss the specific role played by HO-1 in the host response to intracellular pathogens of clinical importance and the potential benefit of targeting HO-1 as a host-directed therapeutic strategy for improving treatment of the diseases they induce.

## 2. Mechanisms of Induction of HO-1 Expression

HO-1 is constitutively expressed at high levels in the liver and spleen reticuloendothelial systems, especially in macrophages that engulf senescent red blood cells and promote, through the action of HO-1, the recycling of iron from heme derived from hemoglobin [27]. Heme can also originate from degradation of other heme containing proteins such as myoglobin, cytochromes and several peroxidases, catalases and oxidases [28], or can be internalized from the extracellular compartment, where free heme and hemoglobin molecules are scavenged by the acute phase proteins hemopexin and haptoglobin respectively and then endocytosed via interaction with the receptors CD91 (heme/hemopexin complex) and CD163 (hemoglobin/haptoglobin complex) [29,30]. Heme is transported from the phagolysosomes to cytosol by the heme responsive gene-1 (HRG-1 or SLC48A1) transporter [31,32] and later to the nucleus where it can directly induce HO-1 expression by playing a pivotal role in a sequence of events that culminate in HMOX1 gene transcription as described below. The mechanisms involved in its transport to the nucleus, however, are not completely understood, although there is evidence identifying the enzyme biliverdin reductase as a mediator in this process [33].

The HMOX1 gene contains in its promoter region a motif known as Maf Recognition Element (MARE) or Antioxidant Response Element (ARE), which is recognized by a dimer composed of the BTB and CNC Homology 1 (BACH1) transcription factor together with one of 3 small Maf oncogenic proteins (MafF, MafG or MafK). Under normal conditions, the BACH1-Maf dimer remains bound to the ARE motif in HMOX1 gene promoter and acts as a repressor of its transcription [34]. However, BACH1 has a heme-binding region, which upon interaction with heme molecules present in the nucleus, promotes dissociation of BACH1 from the Maf protein and from the ARE motif in the HO-1 gene promoter. This event is followed by translocation of BACH1 molecule to the cytoplasm where it undergoes ubiquitination and further degradation by the proteasome [35]. With BACH1 now displaced from the nucleus, Maf forms a dimer with the Nuclear Factor Erythroid 2-Related Factor 2 (NRF2) transcription factor, which then binds to the same ARE region and induces HO-1 gene transcription [36,37].

In order to induce HO-1 expression, NRF2 must be activated simultaneously with BACH1 inactivation. This process is driven by electrophilic and oxidative stresses, which occur for example when heightened production of reactive oxygen species (ROS) is detected [38,39]. During resting conditions, NRF2 is found in the cytoplasm, associated with the protein Kelch-like ECH-Associated Protein 1 (KEAP1), which promotes NRF2 ubiquitination, resulting in proteasomal degradation of the transcription factor [40]. Upon induction of electrophilic and oxidative stresses, the resulting reactive species will cause modifications in specific cysteine residues present on KEAP1, resulting in inhibition of NRF2 ubiquitination. The transcription factor is then released from KEAP1 and translocated to the nucleus where it will dimerize with small Maf proteins to induce HO-1 gene transcription as described above [41,42,43].

As already mentioned, oxidative stress is a major inducer of HO-1 expression and is a process closely linked with inflammation [44,45]. However, a number of different signaling pathways and transcription factors triggered by inflammatory mediators, danger-associated molecular patterns (DAMPs) and pathogen-associated molecular patterns (PAMPs) have also been reported to trigger HMOX1 transcription [46,47]. Mitogen-activated protein kinase (MAPK), phosphatidylinositol 3-kinase (PI3K), tyrosine kinases and protein kinases (PK) A, B, C and G signaling cascades are commonly activated in response to the above mentioned stimuli and are all involved in the induction of HO-1 expression [48,49,50]. For example, PKC can directly phosphorylate a serine residue located at position 40 on the NRF2 protein, which results in its disruption from KEAP1 thus promoting NRF2 translocation to the nucleus [51]. It has also been reported that NRF2 can be directly phosphorylated at several serine and threonine sites by different MAPKs in vivo. Of note, in that latter case, no effects on NRF2-KEAP1 interaction were observed [52]. Indeed, a number of additional studies have confirmed that MAPK activities can induce NRF2 activation in response to different stimuli [53,54,55,56], however, they leave open the possibility that the effect of these enzymes on NRF2 signaling may be indirect.

It is important to note that there are regions in the HO-1 gene promoter that serve as binding sites for other transcription factors apart from NRF2, whose expression can be induced as a result of activation of the same signaling cascades mentioned above, such as heat-shock factors (HSFs), activator protein-1 (AP-1), nuclear factor-kappa B (NFκB) and hypoxia-induced factor 1 α (HIF1α) [48,57]. These alternative pathways are briefly described below.

In rats, two genomic motifs known as heat shock elements (HSEs) are present in the HO-1 gene promoter that serve as binding sites for HSF and have been shown to play a role in the induction of HO-1 expression in response to hyperthermia [58]. In mouse fibroblasts, deletion of the HSF-1 gene results in marked reduction of HO-1 expression in response to heat shock injury [59]. However, the mechanisms involved in HO-1 induction by the interaction of HSF with HSEs in the HO-1 gene promoter vary among species. For example, HSEs identified in the rat HMOX1 promoter are inactive in humans and mice [48], and therefore, further studies are needed to fully elucidate this putative pathway for HO-1 induction.

The transcription factor AP-1 actually encompasses a family of structurally and functionally related transcription factors composed of dimers made up of members of the Jun (c-Jun, JunB and JunD), Fos (c-Fos, FosB, Fra1 and Fra2), ATF (Atf2, Atf3, Atf4, Atf5, Atf6B, Atf7, BATF, BATF2, BATF3, JDP1 and JDP2) and Maf (c-Maf, MafA, MafB, MafF, MafG and MafK Nrl) DNA-binding protein families [60]. Functional AP-1 binding sites have been identified in promoter regions of mouse, rat and human HO-1 genes [61,62,63] and studies have demonstrated a role for AP-1 in the induction of HO-1 expression in cells from mice, rats and humans [64,65,66]. However, the mechanisms through which AP-1 regulates the induction of HO-1 expression are complex and may involve a cooperative interaction between AP-1 subunits and different transcription factors [66,67,68], including NRF2 [69].

NFκB, similar to AP-1, is a generic term that denotes transcription factors formed by the homo or heterodimerization of components of the Rel family of proteins, namely RelA (p65), RelB, c-Rel, p52 and p50 [70]. During resting conditions, NFκB is found in the cytoplasm in an inactive state bound to the Inhibitor of NFκB molecule (I-κB). Upon cell stimulation, the transcription factor subunits are released from I-κB (which is phosphorylated by the kinase IKK—I-κB kinase—and further degraded by the proteasome) and translocated to the nucleus, where they bind to promoter regions of specific genes and induce their transcription [71]. Functional binding sites for NFκB were identified in the promoter regions of the HO-1 gene. These sites were found to be involved in the induction of rat HO-1 expression in response to phorbol myristate acetate (PMA) in vitro [72] and also in the NOS2-mediated upregulation of HO-1 expression in mouse cardiac tissue [73].

HIFs are a family of transcription factors whose expression is upregulated in response to low oxygen concentrations. They are composed of a constant subunit (HIF1β) and one of three variable α subunits (HIF1α, HIF2α and HIF3α), which provide the name for the whole heterodimer. Oxygen is used as a cofactor by the enzyme prolyl hydroxylase (PHD), which initiates a process culminating in ubiquitination and proteasomal degradation of α subunits. As a consequence, under hypoxic conditions, PHD is held inactive resulting in stabilization of α subunits. This process allows these subunits to dimerize with HIF1β and be translocated to the nucleus to initiate gene transcription [74,75]. HIF1α is by far the best studied of these factors and its expression is highly upregulated during inflammation [76]. Binding sites for HIF1α have been identified in the rat HMOX1 gene approximately 9.5 kilobases upstream of the transcription start site and were shown to be responsible for the induction of HO-1 expression in response to hypoxia in rat aortic vascular muscle cells [77]. Induction of HO-1 transcription in response to direct binding of HIF1α to DNA has also been observed in rat renal medullary interstitial cells as well [78]. Human cells also upregulate HO-1 in response to HIF1α, although direct HO-1 induction by HIF1α binding to the HMOX1 gene was not demonstrated [79].

**Figure 1:**
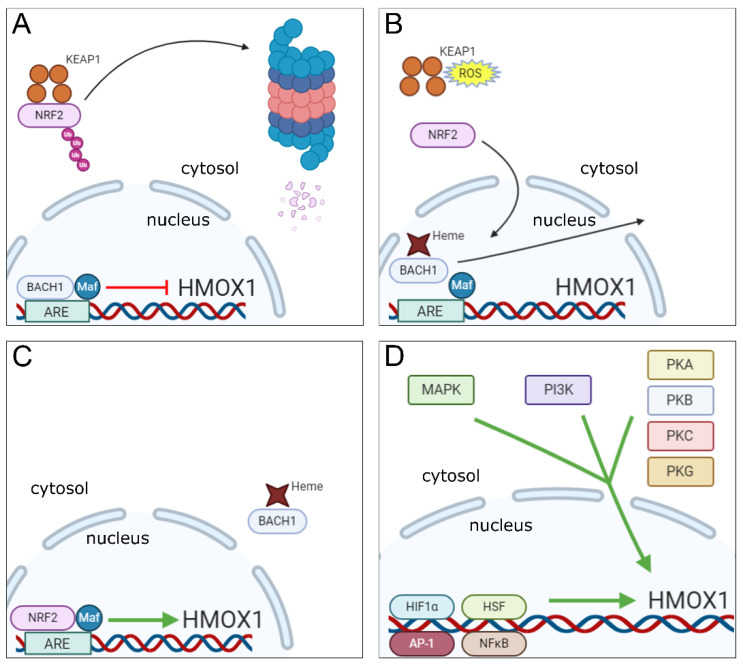
Molecular mechanisms underlying the regulation of HMOX1 gene transcription. (**A**) In homeostatic conditions, NRF2 is found in the cytosol of cells bound to KEAP1, which promotes ubiquitination of NRF2 that is further degraded in proteasomes. In the nucleus, the transcription factor BACH1 remains bound in association with a small Maf protein to the ARE region in HMOX1 gene promoter repressing its transcription. (**B**) Under stress conditions, binding of heme molecules to BACH1 in the nucleus promotes its displacement from the small Maf protein and ARE motif in the HMOX1 gene promoter, while in the cytosol, ROS induce changes in KEAP1 cysteine residues that promote the release of NRF2 and prevents its ubiquitination and degradation. (**C**) NRF2 is translocated to the nucleus, where it associates to a small Maf protein and binds to the ARE motif in HMOX1 gene promoter inducing its expression, while heme-bound BACH1 is excluded from the nucleus. (**D**) Signaling cascades involved in the induction of HMOX1 gene expression (MAPK, PI3K and PKA, B, C and G—cytosol) and transcription factors to which recognition motifs were identified in the HMOX1 gene or that were shown to induce HO-1 expression (HIF1α, HSF, AP-1 and NFκB—nucleus).

The existence of multiple pathways for triggering and repressing HO-1 transcription (summarized in Figure 1) is consistent with a central role for the enzyme in responding to a wide variety of inflammatory stimuli and within a range of different host cell types.

## 3. Pro-Oxidant and Proinflammatory Effects of Heme

Free heme can be found intracellularly as part of the labile heme pool used for the synthesis of hemeproteins. However, excessive cytosolic free heme accumulation can become a proinflammatory stimulus and induce oxidative stress [80]. Hemoglobin and several other hemeproteins can serve as a source of extracellular heme in episodes of hemolysis and necrotic cell death that release heme into the extracellular environment and circulation [81].

The toxicity of free heme stems in great part from its pro-oxidant activity. The iron atom contained in the porphyrin ring from heme molecules can catalyze the generation of hydroxyl-radicals (OH·) from hydrogen peroxide, a process known as the Fenton reaction [82]. It can also be converted into ferryl (Fe^4+^) and perferryl (Fe^5+^) species [83] that rapidly react with organic hydroperoxides (ROOH) to yield highly reactive alkoxyl (RO) and peroxyl (ROO) radicals [84,85] that induce lipid peroxidation and amplify the formation of more peroxyl radicals [86]. More recently, it was demonstrated that heme could induce cell death by ferroptosis, a process involving iron-mediated oxidative stress and cell membrane lipid peroxidation [87,88]. Heme also promotes formation of ROS [89,90]. Together, these highly reactive heme stimulated products can damage nucleic acids, protein and lipid structures in cells [91].

The proinflammatory effects of heme are in turn derived from its pro-oxidant effects and also from its action as a DAMP. Heme molecules can stimulate TLR4 in macrophages, inducing the production of TNF though activation of the MYD-88 pathway [92]. Interestingly, recent evidence indicates that this response is initiated not by direct binding of heme to TLR4 but to its signaling partner MD-2 [93]. The TNF production stimulated by this pathway is temporally associated with a concomitant but independent heme induced production of ROS. Combined, TNF and ROS can trigger death of inflammatory macrophages by necroptosis, a pathway dependent on the activity of the kinases RIPK1 and RIPK3 [94]. Heme has also been shown to induce the activation of the NLRP3 inflammasome resulting in increased secretion of the active form of IL-1β in LPS-primed macrophages [95,96]. Heme can also trigger TLR4 and NLRP3 in endothelial cells resulting in increased expression of adhesion molecules (ICAM-1, VCAM-1 and E selectin) [97,98,99] and ROS-dependent IL-1β production [100], respectively. In neutrophils, heme is able to induce chemotaxis [89,101] and the formation of neutrophil extracellular traps (NET) [102] although the precise mechanisms involved are complex and not fully elucidated.

## 4. Modulation of Oxidative Stress, Immune Responses and Inflammation by HO-1

Heme oxygenase-1 is well known for its anti-inflammatory and immunomodulatory properties, which have been clearly demonstrated in a number of studies. Pharmacological induction of HO-1 or strategies of overexpression of the enzyme gene have been proven to confer host protection in various inflammatory and autoimmune diseases [103]. On the contrary, HO-1 deficiency leads to systemic chronic inflammation in mice [104], while in humans, in the few cases in which this condition was detected, the patients presented heightened sensitivity to oxidative stress, intravascular hemolysis and dysregulation in iron homeostasis along with evidence of kidney, liver and endothelial inflammation [105,106,107].

**Figure 2:**
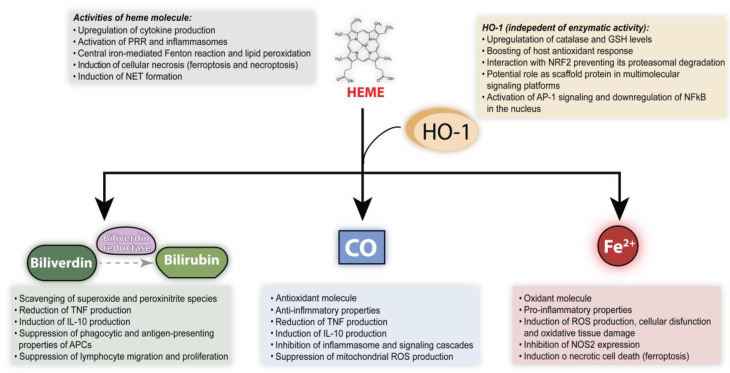
Summary of anti vs pro-oxidant/inflammatory properties of heme, HO-1 and products of heme degradation.

The simple decrease in intracellular heme levels resulting from its degradation by HO-1 is per se an anti-inflammatory consequence. However, the individual products of heme catalysis also play direct roles in the modulation of inflammation and immune responses [108]. Additionally, some studies have demonstrated that HO-1 can perform functions that are independent of its enzymatic activity and play important roles in the regulation of oxidative stress [109]. This evidence is reviewed in details below and summarized in Figure 2.

### 4.1. Carbon Monoxide

CO is a gaseous product of heme degradation by HO-1 that acts as a signaling molecule in a vast array of cell types such as those from the nervous, circulatory, digestive and immune systems [110]. CO has been shown to downregulate the production of cytokines by various leukocyte populations and to be the major player in the immunomodulatory activity of HO-1 in some models of inflammation, such as LPS-induced endotoxemia, hyperoxia and ischemia/reperfusion-induced lung injury and diabetes/hyperglycemia [111].

In vitro, exposure of RAW 264.7 macrophages to CO gas resulted in impaired TNF production in response to activation of TLR2, 4, 5 and 9 [112]. Similarly, in vivo administration of CO by inhalation resulted in enhanced resistance to endotoxemia induced by LPS injection and this protection was associated with decreased p38 MAPK-dependent production of TNF, IL-1β and CCL3 along with enhanced production of anti-inflammatory IL-10 by macrophages [113]. In addition to p38K, CO has been found to induce anti-inflammatory activity by interfering with multiple signaling pathways including ERK MAPK, JNK MAPK, NF-κB and AP-1 [114,115,116].

A variety of studies have also identified a role for CO in the regulation of inflammasome activity. Treatment of bone marrow derived macrophages with CO gas results in inhibition of NLRP3 inflammasome and caspase-1 activation thereby decreasing the secretion of mature IL-1β and IL-18 and production of mitochondrial ROS in response to inflammasome-activating stimuli [117]. CO administration by treatment with the CO donor molecule CORM2 (tricarbonyldichlororuthenium(II)) suppressed NLRP3 inflammasome activation in the human monocytic cell line THP-1 [118], while treatment with the donor CORM3 (tricarbonylchloro(glycinato)ruthenium) inhibited the activation of caspase-1 and production of mature IL-1β by the human monocytic cell line U937 in response to endoplasmic reticulum stress [119]. In vivo, CORM2 treatment prevented NLRP3 activation in an experimental model of LPS-driven acute lung injury [120], while CORM-3 administration resulted in decreased IL-1β production in mice with streptozotocin-induced diabetes [121] and suppressed NLRP3 activation in cardiac fibroblasts from mice with LPS-induced sepsis resulting in improved cardiac function [122].

The use of CO inhalation or administration of CO donors also confers protection against tissue damage in experimental models of autoimmune diseases in which lymphocytes play an important role in pathogenesis. This effect has been observed in murine lupus [123,124], experimental models of arthritis [125,126] and experimental autoimmune encephalomyelitis (EAE) [127].

### 4.2. Biliverdin, Biliverdin Reductase and Bilirubin

Biliverdin (BV) is a product of heme degradation by HO-1, which is subsequently converted to bilirubin (BR) by the activity of the enzyme biliverdin reductase (BVR). Biliverdin and bilirubin display important anti-inflammatory effects and exhibit potent antioxidant properties, acting as direct scavengers of superoxide (O2^−^) and peroxynitrite (ONOO^−^) species [128,129]. Biliverdin treatment resulted in increased survival of rats submitted to a model of endotoxemia by LPS injection, which was associated with milder lung inflammation, decreased systemic IL-6 levels and enhanced IL-10 secretion by macrophages [130]. BV has also been shown to inhibit TNF-induced NFκB activity [131] and suppress proinflammatory cytokine production. The mechanisms underlying this phenomenon potentially involve downregulation of LPS-induced complement receptor 5a (C5aR) expression in macrophages through the mTOR signaling pathway [132].

Interestingly, many of the anti-inflammatory effects attributed to BV are in fact mediated by BVR. Besides acting as an enzyme that catalyzes the reduction of BV to BR, BVR also plays a role both as a receptor triggering intracellular signaling cascades upon binding to BV, and as a transcription factor migrating to the nucleus and binding to promoter regions of several genes and regulating their transcription [108,133].

The induction of IL-10 production by BV treatment in LPS-activated macrophages was found to be dependent on the activity of BVR present on the cell membrane. BV activates a tyrosine kinase domain in BVR that subsequently initiates a signaling cascade through the PI3K-Akt pathway, triggering IL-10 production [129,134]. S-nitrosylation of BVR in conditions of cellular nitrosative stress promotes the translocation of the enzyme to the nucleus, where it can directly bind to the TLR4 gene suppressing its transcription [135]. BVR has also been shown to induce the transcription of AP-1-regulated genes, which are involved in the expression of antioxidant genes such as HMOX1 [136,137].

In addition to BV and BVR, bilirubin (BR) has also been demonstrated to possess immunoregulatory activity. In the innate immune response, complement activation, for example, is blocked by BR, which attenuates the binding of antibodies to C1q and of C3 to sensitized cells [138]. In addition, the expression of Fc receptors and the phagocytic and antigen-presenting properties of peritoneal macrophages are suppressed by BR [139]. Similarly, neutrophil ROS-dependent antimicrobial function [140] and phagocytic [141] and chemotactic [142,143] activities are also inhibited by BR. BR has also been shown to regulate chemotaxis and cell migration, reducing lymphocyte adhesion to microvessels in vivo [144] and repressing the expression of E-selectin, VCAM-1 and ICAM-1 in endothelial cells [145].

In addition to suppressing innate immunity, BR can also interfere with adaptive immune responses impairing IL-2 production and T cell proliferation in response to polyclonal stimulation [146]. Inhibition of T cell proliferation and IL-2 production by BR was also observed in a second study together with suppression of IFNγ production and induction of apoptosis in activated T cells. In the same article, the authors demonstrated that BR treatment of mice with EAE mice attenuates disease symptoms due to its immunomodulatory actions [147]. Similarly, in an experimental model of colitis, BR administration was found to confer protection against tissue injury as a consequence of the induction of IL-10 production by CD4^+^ T cells. The same authors demonstrated that BR treatment of human Th17 cells upregulates expression of FOXP3 and induces an aryl hydrocarbon receptor (AHR)-dependent increase in CD39 ectonucleotidase, both of which have immunosuppressive effects on this CD4^+^ T cell subset [148]. BR was further shown to induce PDL1 expression in murine macrophages, thereby promoting the expansion of FoxP3^+^ regulatory CD4^+^ T cells in coculture experiments [149].

### 4.3. Iron

In contrast to CO and BV, which typically are immunosuppressive in their effects, iron, the third product of HO-1 induced heme degradation is usually thought of as proinflammatory [150]. Ferrous iron released from heme is rapidly bound by the highly efficient iron storing protein ferritin, which can accommodate nearly 4500 iron atoms in an inert form converting them to the ferric state [151]. Fe^2+^ ions not captured by ferritin remain free in the cell cytoplasm or loosely bound to proteins thus constituting the labile iron pool, which as described earlier promotes the formation of hydroxyl-radicals (OH·) through the Fenton reaction [82] and can also mediate formation of alkoxyl (RO·) and peroxyl (ROO·) radicals after conversion to ferryl (Fe^4+^) and perferryl (Fe^5+^) species [83,84,85].

Despite its well-known antioxidant effects, HO-1 activity, when upregulated, can act in the opposite direction inducing intracellular labile iron accumulation leading to ROS production [152,153], effects that were shown to contribute to the cellular dysfunction observed in LPS-induced endotoxic shock [154]. Induction of HO-1 expression by hemin or genetically induced HO-1 overexpression was found to induce ferroptosis, a necrotic form of cell death dependent on iron accumulation and peroxidation of cell membrane lipids [155]. Heme-derived iron bound to ferritin can also be displaced in some situations, resulting in the release of chemically active ferrous iron in the cytosol thereby promoting oxidative tissue damage particularly in situations of iron overload [156,157,158].

However, iron accumulation can also play an immunosuppressive role since labile ferrous iron has also been shown to inhibit the expression of NOS2, an enzyme important for host antimicrobial defense [159,160,161]. Thus, iron accumulation has been shown to repress IFNγ-induced NOS2 expression in one study by preventing the binding of the NF-IL6 (C/EBPβ) transcription factor to the promoter region of the NOS2 gene [162] and in a second study involving *Salmonella typhimurium* infection by inducing the production of the anti-inflammatory cytokine IL-10 [163].

In specific circumstances, iron can act indirectly, in this case in a host protective manner, by inducing the expression of ferritin, which in addition to its iron storage role, also displays important antioxidant, anti-inflammatory and immunosuppressive functions [151]. For example, iron mediated induction of ferritin heavy chain expression protects cells in which HO-1 gene was ablated by siRNA from the oxidative damage mediated by hydrogen peroxide [164]. Ferritin can also be found in the nucleus of corneal epithelial cells, in which it decreases DNA double-strand breaks promoted by UV radiation-induced ROS [165,166]. Similarly, overexpression of ferritin light chain in LPS stimulated RAW 264.7 cells resulted in reduced production of TNF, IL-1β and nitric oxide (NO) along with decreased activation of the MAPK and NF-κB signaling pathways [167]. Furthermore, in lymphocytes, the induction of ferritin heavy chain expression is associated with suppression of T cell proliferation and impairment of B cell maturation [168,169].

### 4.4. Functions of HO-1 Independent of Its Enzymatic Activity

Enzymatically active HO-1 is commonly located in the cytoplasm, in association with the endoplasmic reticulum [49]. However, a study published in 2002 demonstrated that transfection of U937 cells with both the wild type or a mutant HO-1 lacking enzymatic activity, resulted in protection of these cells from oxidative stress. The inactive enzyme, however, displayed its antioxidant properties via upregulation of catalase and glutathione levels, while functional HO-1 conferred protection against oxidative stress mainly through generation of bilirubin [170]. It was later shown that a proteolytic cleavage event at the C-terminal portion of the enzyme can generate a 28 kDa protein that is translocated to the nucleus where it activates AP-1, AP-2, Brn-3 and CBF transcription factors while downregulating NF-κB DNA binding capacity and this response is triggered independently of HO-1′s enzymatic activity [171]. Additionally, it was shown that HO-1 engineered to lack catalytic function can activate the HMOX1 gene promoter thereby enhancing production of the enzyme in a mouse fibroblast cell line and revealing a mechanism by which HO-1 amplifies its own expression through a positive feedback loop [172]. Nuclear HO-1 was later found to interact with NRF2, a phenomenon that reduces proteasomal degradation of the transcription factor and stabilizes its expression in the nucleus, resulting in enhanced expression of antioxidant mediators [173].

Another enzymatic-independent function of HO-1 is related to its potential role as a scaffold protein in multimolecular signaling platforms. In silico analyses performed in prostate cancer cells identified numerous possible physical interactions between HO-1 and intracellular proteins involved in DNA transcription, RNA processing and cytoskeleton reorganization [174]. Indeed, the first study in which HO-1 was purified in 1977 described the enzyme obtained from rat microsomes as a 68 kDa protein [3]. We now know that HO-1 has a 32 kDa mass, while that of HO-2 is 36 kDa [15], making it likely that the protein identified in this pioneering study was in fact a complex formed by HO-1 and HO-2 physical interaction, and it was later proposed that this dimerization may be involved in regulation of enzymatic activity [175].

Considering the role of the above mentioned transcription factors in regulating the expression of important immunomodulatory elements, and the potential contribution of HO-1 in the formation of molecular platforms involved in signaling cascades, it is possible that some of the immunoregulatory and anti-inflammatory roles attributed to the enzyme based on the use of mice genetically engineered to be HMOX1 deficient may in fact be related to enzyme-independent functions of the HO-1 protein.

## 5. Role of HO-1 in Infectious Diseases Caused by Intracellular Pathogens

**Figure 3:**
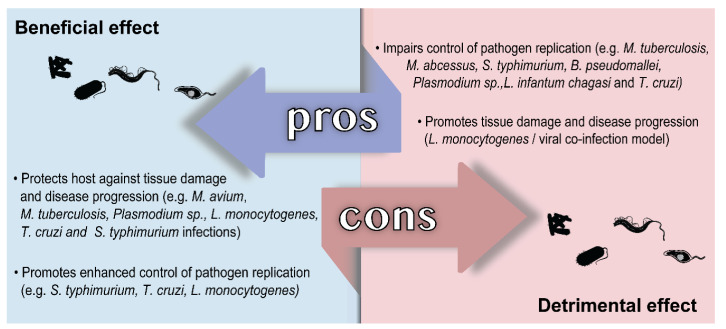
Schematic view summarizing the findings on the beneficial and detrimental roles played by HO-1 in infectious diseases caused by different intracellular pathogens.

In this section, we discussed the role played by host HO-1 and the products of heme degradation in infectious diseases of clinical importance caused by bacterial and protozoal intracellular pathogens. Although there are a number of important diseases caused by intracellular fungi, the role played by host HO-1 in these infections was not studied and therefore was not included. The findings discussed are also summarized for the benefit of the reader in Figure 3.

### 5.1. Diseases Caused by Bacteria

Bacterial infections are among the leading global causes of death according to the World Health Organization (WHO) [176]. Although a wide range of antibiotics have been developed for the treatment of bacterial infections since the discovery of penicillin by Alexander Fleming in 1928 [177], the emergence of antibiotic resistant strains is on the rise and represents a matter of important concern worldwide [178]. Alternative therapies involving immunomodulatory approaches that help the host control bacterial replication more efficiently or dampen the associated immunopathology driven by inflammation are of particular interest as they bypass the problem of antibiotic resistance. Such host-directed therapeutic approaches could also be used adjunctively to improve the efficacy of and/or shorten the course of antibiotic treatment. The role of HO-1 has been extensively studied in several infections caused by intracellular bacteria and represents an interesting target for these novel disease fighting strategies.

#### 5.1.1. Tuberculosis and Other Mycobacterial Infections

Infection with *Mycobacterium tuberculosis* (Mtb) causes tuberculosis (TB), which most commonly targets the lungs but can also affect other organs. TB has been responsible for the largest number of deaths in the world due to an infectious disease for the past two centuries [179]. Mtb is transmitted through the air, when droplets containing bacteria are expelled from the lungs during coughing from an infected individual with cavitary disease. The treatment for TB is long (typically 6–9 months) and employs the use of multiple antibiotics, which can have numerous side effects [180]. For this reason, noncompliance is common among patients resulting in treatment failure, relapse and/or emergence of drug resistant bacteria. Several host molecules have been studied and evaluated as potential targets for the development of new host-directed therapies for TB with the aim of clearing Mtb infection more rapidly and effectively and HO-1 is one of these candidates [181]. In fact, the enzyme has been widely studied in Mtb infection both in vitro and in vivo although there is considerable controversy as to whether the role played by HO-1 in TB is host beneficial or detrimental.

Two studies published in 2008 first demonstrated that Mtb infection induces HO-1 expression in macrophages in vitro and in lung tissue in vivo [182,183]. It was later found that upon infection, macrophages produce ROS in response to ESAT-6 expressed by Mtb, which drives NRF2 activation of HMOX1 gene transcription [184]. In the two seminal papers published in 2008, the authors found that CO released by HO-1-mediated heme degradation acts as a stimulus that induces expression of the Mtb dormancy regulon [182,183]. This set of genes is involved in the conversion of bacilli to a dormant state characterized by extremely low metabolic activity allowing the bacteria to establish a silent latent infection that can last for decades in some individuals later reemerging and causing active disease [185]. Other studies had previously demonstrated that the Mtb dormancy regulon is activated in response to NO [186], a gaseous molecule critical for killing of the bacilli by murine macrophages [187]. For this reason, the above studies led to the hypothesis that HO-1 activity in infected macrophages and subsequent CO release might have toxic or stressful effects on the bacteria similar to NO, inducing dormancy and favoring the establishment of latent infection.

Using a different *Mycobacterium* species, Regev et al. demonstrated that mice genetically deficient in HO-1 are more susceptible to *M. avium* infection displaying increased bacterial dissemination associated with disorganized granuloma formation and abnormal production of CCL2 [188]. An independent study published in the following year yielded similar results and additionally found that the higher susceptibility of HMOX1 gene deficient mice to *M. avium* infection was independent of adaptive immunity and correlated with both enhanced heme-induced necrotic death of macrophages at the sites of infection and increased production of proinflammatory cytokines. The authors also found that mice genetically deficient for HO-1 are more susceptible to *M. tuberculosis* infection, developing higher pulmonary bacterial loads and increased mortality compared to their wild type counterparts [189]. However, it was later demonstrated that HO-1 generated CO is required for IFNγ-induced autophagic killing of Mtb by macrophages, suggesting that the enzyme might play a role in the mechanisms of bacterial control by adaptive immunity as well [190]. Recently, Chinta et al. characterized the expression of HO-1 in lung samples of patients with TB and found that the enzyme is associated with lower ROS and reactive nitrogen species (RNS) production suggesting that HO-1 expression confers protection against oxidative and nitrosative stresses in human TB. Additionally, the authors observed that conditional HO-1 KO mice that lack expression of the enzyme in macrophages are more susceptible to chronic Mtb infection and present enhanced inflammation in lung tissues, although IFNγ production by CD4^+^ T lymphocytes was found to be lower than that of WT Mtb-infected mice [191]. Together, those studies largely supported a host-protective role for HO-1 during Mtb infection.

However, other studies indicated that inhibition of HO-1 activity could promote enhanced bacterial control in vitro and in vivo during mycobacterial infection. For example, Abdalla et al. observed that *M. abcessus* infection induces HO-1 expression in the human monocytic cell line THP-1 via activation of the p38 MAPK pathway. Importantly, the authors found that the treatment of THP-1 cells with the pharmacological inhibitor of HO-1 activity tin protoporphyrin IX (SnPPIX) restricted *M. abscessus* growth in THP1 cells, an effect that was also observed when HO-1 expression was abrogated by siRNA. This enhanced resistance to infection was associated with higher levels of ROS production and lysosomal fusion with infected phagosomes in the absence of HO-1 activity [192]. Similarly, Scharn et al. demonstrated that the inhibition of HO-1 activity by SnPPIX treatment of Mtb-infected human monocyte-derived macrophages results in enhanced control of bacterial replication in vitro [193].

In a related study we used SnPPIX treatment as means of inhibiting HO-1 activity in vivo during Mtb infection in mice. Pharmacological inhibition of the enzyme resulted in a highly significant reduction of pulmonary bacterial loads, which, when performed in conjunction with conventional antibiotic therapy, resulted in accelerated clearance of Mtb infection. Interestingly, this effect was dependent on the presence of T lymphocytes since bacterial loads were unchanged after SnPPIX treatment of mice lacking conventional TCR αβ T cells infected with Mtb [194]. In more recent work, we established that the effect of SnPPIX administration on Mtb infection depends on the production of IFNγ by T cells and NOS2 expression in vivo. We additionally found that SnPPIX mediated inhibition of HO-1 enzymatic activity results in a reduction in intracellular labile iron concentration along with enhanced IFNγ-induced NOS2 expression, NO production and control of bacterial replication. Importantly, supplementation of iron, but not CO or BV to IFNγ-activated Mtb-infected macrophages reverted the effects of enzyme inhibition arguing that induction of HO-1 expression in Mtb-infected cells favors iron accumulation, which in turn impairs NOS2 expression, NO production and control of bacterial replication in response to IFNγ activation [195]. Together, the above findings revealed a host detrimental facet of HO-1 induction in mycobacterial infection that directly contrasts with the beneficial effects initially described for this response.

It is important however to note that most if not all of the studies in which HO-1 expression was associated with protection against mycobacterial infection utilized mice that were deficient in HO-1 protein expression, while those employing pharmacological inhibition of the enzyme’s activity concluded that induction of the HO-1 promotes host susceptibility to the pathogen. These distinct outcomes may reflect the difference between complete absence of the enzyme molecule versus inhibition of its activity. Thus, as discussed above, the HO-1 protein has functions that are independent of its enzymatic activity that might play a role in limiting infection with mycobacteria. Such functions would be absent in HMOX1 gene deficient mice in which expression of the entire protein is lacking, but not in wild type mice treated with inhibitors of its enzymatic activity. Future studies employing mice expressing HO-1 molecules with specific mutations confined to the active site of the enzyme could be used to test this hypothesis.

#### 5.1.2. Salmonellosis

The role of HO-1 has also been investigated in infections with *Salmonella* species and as with mycobacteria, there is no consensus whether the enzyme expression is associated to protection or susceptibility to infection. Most, if not all papers use as a model infection with *Salmonella typhimurium*, a bacterial species that is a major cause of foodborne gastroenteritis [196]. A study published by Zaki et al. in 2009 demonstrated a protective role for HO-1 in a murine model of salmonellosis. The authors found that infection by *S. enterica typhimurium* induces the expression of HO-1 in liver tissue in vivo and macrophages in vitro and this response was found to be dependent on the formation of the GMP derivative 8-nitro-cGMP product of NOS2-mediated NO generation. Inhibition of HO-1 activity by zinc protoporphyrin IX (ZnPPIX) enhanced infection-associated apoptosis in liver cells in vivo and macrophages in vitro, while intracellular bacterial killing was impaired in liver tissue in vivo and RAW 264.7 cells and peritoneal macrophages in vitro upon enzyme activity inhibition. HO-1 ablation by siRNA also resulted in higher apoptotic cell death and poorer control of bacterial replication in peritoneal macrophages [197]. In agreement with these data, Onyiah et al. found that treatment with an HO-1 inducer, cobalt protoporphyrin XI (CoPPIX), protected mice against enterocolitis induced by infection with *S. typhimurium*, resulting in decreased bacterial loads in the lamina propria, mesenteric lymph nodes, spleen and liver. Murine macrophages genetically knocked down for HO-1 were more susceptible to infection by *S. typhymurium* and also *Escherichia coli* and *Enterococcus faecalis*. The enhanced protection to infection promoted by HO-1 expression was associated with increased acidification of lysosomes in response to CO produced by the enzymatic reaction [198].

However, in the opposite direction, Mitterstiller et al. demonstrated that treatment of RAW 264.7 cells with the HO-1 inhibitor ZnPPIX or knockdown of the enzyme expression resulted in reduced survival of *S. typhimurium* upon infection. This outcome was associated with enhanced TNF, ROS and RNS production, NFκB activation and restricted intracellular iron accumulation. The latter effect correlated with increased iron efflux by ferroportin after HO-1 ablation [199], a mechanism that had been previously found to be a major effector restricting *Salmonella* replication in macrophages [200]. One possible explanation of these discrepant findings is that in the in vivo models of infection with *Salmonella* involving long term exposure to the pathogen, the observed detrimental effects of HO-1 inhibition may reflect a dominant requirement for the cytoprotective functions of HO-1 in reducing tissue damage caused by inflammation. Such a requirement would not be evident in the situation of bacterial growth in vitro.

#### 5.1.3. Listeriosis

Another bacterial disease in which the role of HO-1 has been investigated is listeriosis, caused by *Listeria monocytogenes*. The bacteria are transmitted by contaminated food and typically cause mild, self-limiting gastroenteritis and fever. However, in elder people, newborns and immunocompromised individuals, *L. monocytogenes* infection can cause severe sepsis, meningitis or encephalitis, sometimes resulting in death, while infection of pregnant women can lead to spontaneous abortion [201].

Tzima et al. demonstrated that mice with conditional deletion of HO-1 in cells that express lysozyme M (mostly macrophages and neutrophils) are more resistant to infection with *L. monocytogenes* in a murine model of viral–bacterial coinfection. The authors infected wild type and HO-1 conditional KO mice with *L. monocytogenes* resuspended in a buffer solution containing poly I:C and found that wild type mice had a higher mortality rate and decreased number of liver abscesses when compared to HO-1 conditional KO mice. HO-1 conditional KO mice presented lower levels of circulating IFNβ and the authors attributed their increased resistance to infection to a reduction in IFNβ-induced cell death of infected cells, which in wild-type animals promotes bacterial survival and spread [202].

In the opposite direction, a study identified a host protective role for HO-1 expression in *L. monocytogenes*-infection during pregnancy. The authors found that infection of trophoblast giant cells with the bacteria resulted in suppression of HO-1 expression and that treatment of *L. monocytogenes*-infected pregnant mice with the HO-1 inducer CoPPIX substantially decreased infection-induced abortion [203]. More recently, Wang et al. found that the suppression of NRF2 nuclear translocation and consequent reduction of HO-1 expression in response to Tim-3 receptor signaling in macrophages are associated with enhanced susceptibility to *L. monocytogenes* infection in vitro and in vivo [204]. The latter studies thus suggest that HO-1 expression favors the control of bacterial replication during *L. monocytogenes* infection. The contrasting results obtained by Tzima et al. may relate to the different experimental conditions employed since the coadministration of poly I:C was critical for revealing the role of HO-1, which in this situation was important for the modulation of IFNβ production driven by poly I:C activation [202].

#### 5.1.4. Melioidosis

*Burkholderia pseudomallei* is an environmental bacterium found in the soil of endemic regions, which can infect humans and cause melioidosis, a disease often characterized by severe pneumonia with a mortality rate of around 40% [205]. Treatment of *B. pseudomallei*-infected mice with the HO-1 inducer CoPPIX impaired the control of bacterial replication in several organs in vivo, and in macrophages in vitro, while HO-1 gene knockdown improved the restriction of intracellular pathogen replication by these cells. The authors identified CO as the product of heme degradation by HO-1 involved in this outcome, since the treatment of infected mice in vivo or macrophages in vitro with a CO donor molecule (CORM2), impaired the control of bacterial replication by the host [206]. Additionally, suggesting a detrimental role for HO-1 in melioidosis, Nithichanon et al. reported that the enhanced susceptibility of patients with beta-thalassemia to *B. pseudomallei* infection is associated with an excess of hemin, which induces expression of high levels of HO-1 and consequently impairs their capacity to control infection [207]. Together, these studies argue that the induction of HO-1 expression in *B. pseudomallei* infection impairs the control of bacterial replication and thus plays a detrimental role in host resistance to infection.

### 5.2. Diseases Caused by Protozoan Parasites

Diseases caused by protozoan parasites are a major cause of death worldwide and, with the exception of malaria, to this day remain largely neglected [208,209,210]. Treatment for these infections is complicated by several factors. Many of the drugs used induce important side effects and the same pathogen can cause different manifestations of disease that require distinct therapeutic strategies [211]. In common with other infections, collateral tissue damage due to inflammation is an important aspect of diseases caused by protozoan parasites and therefore, modulation of inflammatory and immune responses represent a logical approach for the design of novel therapies [209]. In that regard, the role of host HO-1 has been evaluated in several studies utilizing experimental models of infection and diseased patients.

#### 5.2.1. Malaria

Malaria is the leading cause of mortality due to a protozoan parasite, with an estimated 405 thousand deaths in 2018 [212]. In humans, the disease is caused by different species of *Plasmodium*, mainly *P. falciparum*, *P. vivax*, *P. malariae*, *P. ovale* and *P. knowlesi*, which are transmitted to humans through the bites of mosquitoes of the *Anopheles* genus. The most common symptom of malaria is the occurrence of periodic fever episodes every 48–72 h, however, more severe forms of the disease include pulmonary, kidney and especially neurologic manifestations [208].

Malaria was one of first infectious diseases in which the role of HO-1 was evaluated in detail. In 2007, Pamplona et al. used an experimental model of cerebral malaria in which mice were infected with *P. berghei* ANKA and found that HO-1 expression protected mice from the development of cerebral malaria. The authors observed that BALB/c mice were more resistant to the development of cerebral malaria than C57BL/6 animals and that this outcome was associated with higher induction of HO-1 expression in BALB/c compared to C57BL/6 mice upon infection. Pharmacological inhibition of HO-1 activity or deletion of the HMOX1 gene in BALB/c mice resulted in increased mortality due to cerebral malaria, while induction of HO-1 expression in infected C57BL/6 mice by the administration of CoPPIX protected the animals from cerebral malaria resulting in enhanced survival. The authors further observed that CO administration was able to mimic the effects of pharmacological HO-1 induction in C57BL/6 mice, which appeared to be due to the ability of CO in inhibiting the release of heme from hemoglobin. The protective effect was associated with a reduction in blood–brain barrier disruption, neuroinflammation and CD8^+^ T cell infiltration in neural tissue [213]. Pharmacological induction of HO-1 expression and CO administration were also both found to play a host protective role against acute lung injury induced by infection with *P. berghei* ANKA in mice [214,215].

In further work, HO-1 was shown to confer host protection against *P. chabaudi chabaudi* infection in mice. In this study, the authors found that HMOX1 gene deletion resulted in the development of lethal hepatic failure upon *P. chabaudi chabaudi* infection. They further demonstrated that HO-1 expression and its antioxidant function counteracted the pro-oxidant effects of free heme, which sensitizes hepatocytes to undergo TNF-induced cell death [216]. Importantly, in all of the above studies with murine malaria, the beneficial effects of HO-1 expression were predominantly related to protection against inflammation and tissue damage, while little or no effect was observed on parasite survival/replication.

In contrast, while Epiphanio et al. found that *P. berghei* and *P. yoelii* infection of BALB/c mice induces an enhancement in hepatic HO-1 expression, HMOX1 gene deletion was found to confer protection against liver infection with these two species of *Plasmodium*. Similarly, HO-1 overexpression or treatment of wild-type mice with CO or biliverdin resulted in increased hepatic parasite loads, which in turn were reduced in the livers of infected, untreated HO-1-deficient animals. The protective effect of HO-1 depletion was associated with increased infiltration of inflammatory cells in the liver and heightened expression levels of CCL2, CCL3, TNF and IL-12 [217]. In humans infected with *P. falciparum*, HO-1 expression also appears to be host detrimental. Thus, analyses of single nucleotide polymorphisms indicated that patients who carry a polymorphism in the HMOX1 gene promoter that results in heightened levels of HO-1 expression develop more severe forms of disease and have a higher mortality rate than those carrying the allele associated with lower levels of HO-1 expression [218].

Although there are contradictory results about the role of HO-1 in malaria, it is important to mention that this is a complex disease, involving different species of *Plasmodium* that can have distinct manifestations. Together the studies reviewed above suggest that the beneficial vs. detrimental effects of HO-1 expression in malaria depend on both the infecting parasite and disease affected tissue and thus may be difficult to generalize.

#### 5.2.2. Leishmaniasis

Leishmaniasis is caused by the infection with protozoan parasites from the genus *Leishmania*. The pathogens are transmitted to humans by the bites of sandflies from the genus *Lutzomyia* in the Americas and *Phlebotomus* in Africa, Europe and Asia. There are three major forms of leishmanial disease: cutaneous, mucocutaneous and visceral. Some of the most common species that cause human infection are: *L. braziliensis*, *L. amazonensis*, *L. mexicana*, *L. guyanensis* and *L. infantum chagasi* in the new world and *L. major*, *L. tropica*, *L. aethiopica* and *L. donovani* in the old world [219]. The visceral form, which is the most dangerous manifestation, is responsible for around 20–40 thousand deaths every year [220].

Pham et al. identified in 2005 that the infection of macrophages with *L*. *mexicana pifanoi,* which causes cutaneous disease, induces enhancement of HO-1 expression. Pharmacological inhibition of the enzyme activity by tin mesoporphyrin (SnMP) treatment resulted in higher ROS production while administration of the HO-1 inducer CoPPIX led to a decrease in ROS levels. However, the role of the enzyme in the control of parasite replication was not assessed in this study [221]. Infection of murine macrophages with a species that causes visceral disease, *L. infantum chagasi*, was also shown to induce HO-1 expression. Treatment with the enzyme inducer CoPPIX impaired the control of pathogen replication while HMOX1 gene deficiency resulted in reduced numbers of parasites per macrophage. Furthermore, the authors found that the circulating levels of HO-1 were higher in human patients with visceral leishmaniasis compared to healthy controls and demonstrated that human macrophages display enhanced parasite replication when HO-1 expression is pharmacologically induced by CoPPIX treatment [222]. More recently, it was demonstrated that HO-1 gene silencing confers increased protection against *L. donovani* infection in a mouse macrophage cell line. This effect was associated with higher production of ROS, IL-12 and TNF, along with enhanced interaction of TLR4 with MyD88 and TRIF adaptors and increased NFκB and IRF3 activation. The authors further showed that treatment with a CO donor molecule could revert these effects, arguing that CO is the product of heme degradation by HO-1 responsible for the anti-inflammatory actions of the enzyme during infection. In addition, this study also demonstrated that in vivo treatment of *L. donovani*-infected BALB/c mice with the HO-1 inhibitor SnPPIX results in a reduction in parasite loads in liver and spleen along with increased production of IL-12 and TNF [223]. Thus, together these studies demonstrated a host detrimental effect of HO-1 expression during leishmaniasis.

Dogs play an important role in the urban life cycle of leishmaniasis and act as the main reservoirs for the parasites in this niche [224]. Almeida et al. demonstrated that infection of dog macrophages with *L. infantum chagasi* induces HO-1 expression. They further observed that treatment of cells with the HO-1 inducer CoPPIX results in impaired control of parasite replication associated with decreased superoxide, ROS and NO production. In the opposite direction, treatment with the HO-1 enzyme inhibitor SnPPIX resulted in a lower infection rate and decreased production of the anti-inflammatory cytokine IL-10 [225].

The above-mentioned studies clearly indicate that the induction of HO-1 expression during infection with different species of *Leishmania* parasites is detrimental for the host and impairs the control of pathogen replication. Moreover, it was recently demonstrated that exposure of human and mouse skin to sandfly bites or intradermal inoculation of vector salivary gland extract in mouse ears induce HO-1 expression in inflammatory cells recruited to the bite/inoculation sites [226]. This phenomenon may therefore favor the establishment of *Leishmania* infection, given the detrimental role of HO-1 expression for restriction of parasites by host cells.

#### 5.2.3. Chagas Disease

Chagas disease or American trypanosomiasis is caused by infection with the protozoan parasite *Trypanosoma cruzi*, which is transmitted by the hematophagous triatomine insect species *Triatoma infestans*, *Rodhinus prolixus, Panstrongylus megistus, T. dimidiata* and *Rhodnius pallescens* [227,228]. Chagas disease is estimated to affect approximately 11 million people around the globe [229]. While the majority of infected individuals remain asymptomatic, those who manifest the disease can develop highly debilitating chronic cardiomyopathy or megacolon [230].

There are only two studies in which the role of HO-1 during *T. cruzi* infection was evaluated and they present contrasting results. Paiva et al. found that treatment of *T. cruzi*-infected mice with the HO-1 inducer CoPPIX resulted in reduced parasite load, while an increase in pathogen burden occurred when the HO-1 inhibitor SnPPIX was administered. The same effects were observed in vitro in *T. cruzi*-infected macrophages, in which the protective effects of CoPPIX-dependent HO-1 induction were mimicked by HO-1 gene overexpression. The authors further observed that HO-1 expression induces upregulation of ferritin and ferroportin in macrophages, resulting in reduced intracellular iron and oxidative stress, which normally favor the control of parasite replication by host cells [231].

In contrast, Gutierrez et al. found that inhibition of HO-1 activity in *T. cruzi*-infected mice by the administration of ZnPPIX resulted in reduced parasite numbers in the circulation and tissues. However, infected animals treated with HO-1 inhibitor died earlier than control vehicle-treated mice. On the other hand, administration of hemin, an HO-1 inducer, resulted in higher parasitemia and tissue parasite burden, but survival was not significantly altered in comparison to control animals. The authors further demonstrated that HO-1 inhibition caused an enhancement in cardiac inflammation along with higher expression of IFNγ and TNF. ZnPPIX treatment also resulted in higher production of these cytokines in spleens, accompanied by a reduction on the frequency of regulatory T cells [232].

Although these two studies (both employing the Y strain of *T. cruzi*) present divergent results regarding the role of HO-1 in the host control of parasite replication, both demonstrate that the enzyme plays a role in protecting the host from tissue damage caused by infection-induced inflammation.

#### 5.2.4. Toxoplasmosis

Toxoplasmosis is a disease caused by the infection with the intracellular parasite *Toxoplasma gondii*. Transmission occurs by ingestion of cysts present in the meat of infected animals or oocysts released in the feces of infected felines, which can contaminate food and water. The parasite can infect virtually all worm-blooded animals and approximately one third of the world human population is estimated to be latently infected with *T. gondii*. The infection is usually asymptomatic, however, when occurring during pregnancy it can be transmitted to the fetus and induces spontaneous abortion, hydrocephaly and mental retardation. The infection is also particularly dangerous to immunocompromised individuals, in whom the parasite if left untreated can cause a fatal neurological disease [210].

There is only one published study in which the role of HO-1 was assessed in *T. gondii* infection, which was found to induce enhancement of enzyme expression in lungs of both BALB/c and C57BL/6 mice. HO-1 blockade by ZnPPIX treatment of *T. gondii*-infected mice from both strains resulted in increased parasite burden in lungs, while HO-1 induction by hemin treatment resulted in a reduction of parasite load in lungs of BALB/c mice and in the intestines of C57BL/6 animals [233]. The results of this study therefore support a host protective role for HO-1 in *T. gondii* infection.

## 6. Conclusions

A functional immune system is crucial for the host defense against infectious agents. For intracellular pathogens, in particular, the adequate development of cellular immune responses with participation of T lymphocytes is critical for containing microbial replication [234]. However, often the exacerbated inflammation that follows is detrimental to the host and causes tissue damage that can have a fatal consequence. Therefore, in these cases it is also imperative to keep the development of immune responses against a pathogen in check in order to guarantee the survival of the host [235]. Thus, the ideal response is a mix of resistance to infection with tolerance to infection, which means mounting an adequately regulated immune response that will contain pathogen replication without causing damage to the host.

The antioxidant, anti-inflammatory and immunoregulatory roles of HO-1 activity have been shown to play a host protective role in purely inflammatory and autoimmune conditions, however, as discussed in this review, this is not always the case during infection with intracellular pathogens, since the enzyme can impair the ability of immune cells to contain pathogen replication and dissemination. Nevertheless, in all of the infectious diseases presented here, there is strong induction of HO-1 expression, which in each infection has been shown to play an important role in disease outcome. The beneficial or detrimental effects of the enzyme expression however, are intimately dependent on the particular infecting pathogen and on which tissue is the main site of infection or target of the inflammatory response. For example, during infection with *Plasmodium* species that can cause cerebral malaria, or *T. cruzi*, which can induce life threatening cardiac inflammation, the administration of HO-1 inducing agents can be explored as an alternative to suppress potentially fatal infection-induced inflammation in these vital organs and protect the host. On the other hand, inhibitors of the enzyme activity can be used to boost immune responses and microbicidal activity of infected cells, such as reported for leishmaniasis, melioidosis and, as proposed by our group, for tuberculosis.

Metalloporphyrins are the most well studied compounds that act as HO-1 inducers or inhibitors. Those molecules are heme analogues that contain different metal ions in the center of the porphyrin ring and have been used in many of the studies involving infection with intracellular microorganisms cited in this review (Table 1). The clinical use of these substances, however, has been limited due to some side effects associated with their use, the most important being the development of photosensitivity [236], and to the off-target effect displayed by some of these compounds, which can also interact with heme-containing proteins, such as NOS2, cytochrome p450 and soluble guanylate cyclase, inhibiting their activity [237]. In the case of infections with intracellular pathogens, the off-target effects mentioned above need to be carefully taken into consideration, since these unrelated enzymes could play critical roles in the host resistance to infection, as is the case for NOS2.

Importantly, despite these concerns a wide range of novel HO-1 inducers and inhibitors and drugs that act as donors or scavengers of products of heme degradation are available many of which already have been tested in experimental models and even in some human diseases [238,239,240,241]. This situation together with the ever-growing knowledge regarding the role played by the enzyme in infectious diseases, pave the way for considering HO-1 and the products of its activity as important targets for the development of new therapies for infectious diseases and in particular for those involving pathogens that have proved difficult to treat with conventional antibiotics or readily develop resistance to the same agents. While such HO-1 targeted HDT are likely to be tested and deployed as adjuncts to existing pathogen directed drug treatment, their possible use in certain settings as monotherapies should not be ignored.

## Figures and Tables

**Table 1:** Structures and names of most common HO-1 inhibitors and inducers tested in infections with intracellular pathogens.

Compound Structure	Compound Name and Function	Infections with Intracellular Pathogens in which Compound was Tested
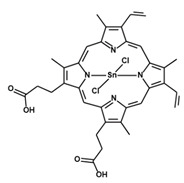	Tin Protoporphyrin IX (dichloride) SnPPIX HO-1 activity inhibitor	*M. abcessus*—improves control of pathogen replication in vitro in THP-1 cells [192]. *M. tuberculosis*—improves control of pathogen replication in vivo in mice [194,195] and in vitro in mouse [195] and human [193] macrophages. *L. donovani*—improves control of pathogen replication in vivo in mice [223]. *L. infantum chagasi*—improves control of pathogen replication in vitro in dog macrophages [225]. *T. cruzi*—impairs control of pathogen replication in mice in vivo and in vitro in macrophages [231].
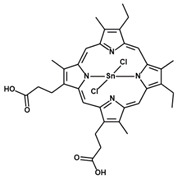	Tin Mesoporphyrin IX (dichloride) SnMP HO-1 activity inhibitor	*L. Mexicana pifanoi*—increases ROS production in vitro in infected mouse macrophages [221].
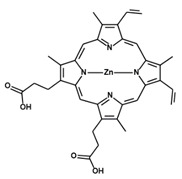	Zinc Protoporphyrin IX ZnPPIX HO-1 activity inhibitor	*S. typhymurium*—impairs control of pathogen replication in mice in vivo and in vitro in peritoneal macrophages and RAW 267 cells [197]. Improves control of pathogen replication in vitro in RAW 267 cells [199]. *P. berghei* ANKA—impairs survival in murine model of cerebral malaria [213]. *T. cruzi*—improves control of pathogen replication in mice in vivo [232]. *T. gondii*—impairs control of parasite replication in mice in vivo [233].
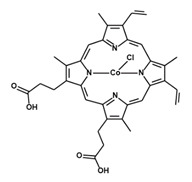	Cobalt Protoporphyrin XI (chloride) CoPPIX HO-1 inducer	*S. typhymurium*—improves control of pathogen replication in mice in vivo [198]. *L. monocytogenes*—decreases the rate of infection-induced abortion in mice [203]. *B. pseudomallei*—impairs control of pathogen replication in mice in vivo and in vitro in macrophages [206]. *P. berghei* ANKA—improves survival in murine model of cerebral malaria [213]. *L. mexicana pifanoi*—decreases ROS production in vitro in infected mouse macrophages [221]. *L. infantum chagasi*—impairs control of pathogen replication in vitro in mouse and human macrophages [222]. *T. cruzi*—improves control of pathogen replication in mice in vivo and in vitro in macrophages [231].
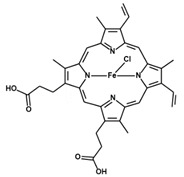	Hemin Ferriprotoporphyrin XI (chloride) HO-1 inducer	*P. berghei* ANKA—decreases inflammatory tissue damage in lungs of infected mice [215]. *T. gondii*—improves control of pathogen replication in mice in vivo [233].

## Abbreviations

Akt: Protein Kinase B; AP-1: Activator Protein-1; AP-2: Activating Protein-2; BACH1: BTB and CNC Homology 1; BR: Bilirubin; BV: Biliverdin; BVR: Biliverdin Reductase; CBF: Core Binding Factor; CCL: Chemokine C-C motif Ligand; CO: Carbon Monoxide; CoPPIX: Cobalt Protoporphyrin IX; DAMP: Danger Associated Molecular Pattern; EAE: Experimental Autoimmune Encephalitis; ESAT-6: 6 kDa Early Secretory Antigenic Target; FOXP3: Forkhead Box Protein P3; GMP: Guanosine Monophosphate; HDT: Host Directed Therapy; HIF: Hypoxia Induced Factor; HO: Heme Oxygenase; HRG-1/SLC48A1: Heme Responsive Gene-1/Solute Carrier Family 48 Member 1; HSE: Heat Shock Element; HSF: Heat Shock Factor; HSP: Heat Shock Protein; ICAM-1: Intracellular Adhesion Molecule-1; IFN: Interferon; IKK: I-κB kinase; IL: Interleukin; I-κB: Inhibitor of Nuclear Factor κ B; KEAP1: Kelch-like ECH-Associated Protein 1; LPS: Lipopolysaccharide; Maf: Musculoaponeurotic fibrosarcoma; MAPK: Mitogen Activated Protein Kinase; MARE/ARE: Maf Recognition Element/Antioxidant Responsive Element; MD-2: Myeloid Differentiation Factor-2; Mtb: *Mycobacterium tuberculosis*; mTOR: Mammalian Target of Rapamycin; MYD-88: Myeloid Differentiation Primary Response 88; NADPH: Nicotinamide Adenine Dinucleotide Phosphate (reduced form); NF-IL6 (C/EBPβ): Nuclear Factor for IL-6 (CCAAT Enhancer Binding Protein β); NFκB: Nuclear Factor κ B; NLRP3: NLR family pyrin domain containing 3; NRF2: Nuclear Factor Erythroid 2-Related Factor 2; NO: Nitric Oxide; NOS2: Nitric Oxide Synthase 2; PAMP: Pathogen Associated Molecular Patten; PDL1: Programmed Death Ligand-1; PHD: Prolyl Hydroxylase; PI3K: Phosphoinositide 3-Kinase; PK: Protein Kinase; Poly I:C: Polyinosinic-polycytidylic Acid; RIPK: Receptor Interacting Serine/Threonine Protein Kinase; RNS: Reactive Nitrogen Species; ROS: Reactive Oxygen Species; SnMP: Tin Mesoporphyrin IX; SnPPIX: Tin Protoporphyrin IX; TB: Tuberculosis; TLR: Toll Like Receptor; TNF: Tumor Necrosis Factor; VCAM-1: Vascular Cell Adhesion Protein-1; ZnPPIX: Zinc Protoporphyrin.
